# Certainty and safe consequence responses provide additional information from multiple choice question assessments

**DOI:** 10.1186/s12909-017-0942-z

**Published:** 2017-06-28

**Authors:** M.J. Tweed, S. Stein, T.J. Wilkinson, G. Purdie, J. Smith

**Affiliations:** 1 Department of Medicine, University of Otago Wellington, PO Box 7343, Wellington, 6242 New Zealand; 2 Distance Learning, University of Otago, Dunedin, New Zealand; 3 Medical Education Unit, University of Otago Christchurch, Christchurch, New Zealand; 4 Deans Department, University of Otago Wellington, Wellington, New Zealand; 5 College of Education, University of Otago, Dunedin, New Zealand

**Keywords:** Assessment, Certainty, Consequence, Multiple choice question, Safety

## Abstract

**Background:**

Clinicians making decisions require the ability to self-monitor and evaluate their certainty of being correct while being mindful of the potential consequences of alternative actions. For clinical students, this ability could be inferred from their responses to multiple-choice questions (MCQ) by recording their certainty in correctness and avoidance of options that are potentially unsafe.

**Methods:**

Response certainty was assessed for fifth year medical students (*n* = 330) during a summative MCQ examination by having students indicate their certainty in each response they gave on the exam. Incorrect responses were classified as to their inherent level of safeness by an expert panel (response consequence). Analyses compared response certainty, response consequence across student performance groupings.

**Results:**

As students’ certainty in responses increased, the odds they answered correctly increased and the odds of giving unsafe answers decreased. However, from some ability groups the odds of an incorrect response being unsafe increased with high certainty.

**Conclusions:**

Certainty in, and safeness of, MCQ responses can provide additional information to the traditional measure of a number correct. In this sample, even students below standard demonstrated appropriate certainty. However, apart from those scoring lowest, student’s incorrect responses were more likely to be unsafe when they expressed high certainty. These findings suggest that measures of certainty and consequence are somewhat independent of the number of correct responses to MCQs and could provide useful extra information particularly for those close to the pass-fail threshold.

## Background

A clinician may consider alternative diagnoses and/or treatments for a patient. The clinician will weigh up the need to seek further information based on self-assessed ability and on the potential benefits and harm to the patient. This weighing up may not lead to the need to check resources if potential benefits are near equivalent and potential for harm minimal. In contrast, if options have potential for harm, and/or the clinician is uncertain, that clinician may decide to defer to others or seek extra information. As such, certainty, safety and knowledge could be independent but intertwined factors.

Effective self-monitoring, evaluation of performance in the moment, along with self-regulation and self-assessment are bases of current professional practice [1–7]. Clinicians consider their decisions regarding whether they need to seek assistance, or additional information, in confirming their decisions [8]. Self-monitoring of a clinical decision is manifest by considering, “when to look it up” or “defer to others” and is an example of reflection-in-action of daily practice [1, 6, 7, 9]. Factors influencing these considerations include beliefs about capabilities, consequences and alternatives [10–12]. In clinical practice, an inappropriate degree of certainty in a decision, or not considering consequences of a decision, holds potential for adverse outcomes and patient harm [13–15]. There is probably an underestimate of the patient harm that results from a failure to make the correct diagnosis and management [16]. In hospital practice, 10% of adverse events are contributed to by diagnostic error, with 74 to 96% of these errors including some cognitive factors [17].

Those who are less able have been found also to be less aware of their deficiencies, resulting in an inappropriately high certainty in their correctness of decisions [18–20] and higher incidence of error [21, 22]. Some previous studies, that linked lower performance scores with lower awareness and with undue certainty, were limited as they used norm-referenced scores to define ability groups [23, 24].

Concern has also been raised about those who have sufficiently good examination performance to score above a pass threshold in assessments but whose incorrect responses are considered dangerous, possibly demonstrating a lack of consideration of consequences of decisions [25–28]. Thus, getting enough correct responses to pass is not offset by noting their incorrect dangerous responses. Making a guess, while not considering the consequences, is not appropriate in clinical practice [29]. Studies raising these concerns are limited in that the results are mostly from retrospective analyses of responses, when the examinees were not informed that this analysis would be undertaken. These limitations are significant because instructions relating to unsafe responses can alter response patterns [30]. Errors of commission are worse than an acceptance of no knowledge [31], but if the instruction of the assessment, either explicit or implied, is “guess if you don’t know to increase your score”, the meaning of these apparent errors cannot be evaluated.

### Planning this study

This study addresses the question of whether, when assessing healthcare professionals or students, there is potential value in considering their demonstration of certainty in responses to questions related to decision-making situations and avoidance of responses that would be potentially unsafe in practice [32–34].

The development of an assessment system that could include certainty in, and safeness of, responses, has proven possible in the context of a research project [32, 34]. However student response behaviour within a research project is likely to differ from that in a summative assessment setting that is consequential to students. This research builds on prior work by combining certainty and safety responses in assessment with summative consequences and by making comparisons with the concurrent measures of assessment performance, as judged by number of correct responses, including a passing standard.

The nature of the relationship, or lack of it, between the number of assessment items answered correctly, as a surrogate for ability, and appropriate certainty and/or consequence when incorrect needs exploration in a prospective manner and in summative assessment. This has not been undertaken before and is the focus of this study.

This research set out to address three questions:

1. What is the relationship between response certainty and odds of correctness?
2. What is the relationship between response certainty and the odds of potentially adverse consequence?
3. How do these relationships of certainty with correctness and consequence vary with ability groupings, derived from the number correct on the assessment?

## Method

### Setting

The Medicine degree course at the University of Otago is a six year course that is divided into four distinct periods. The first is a common health science year. This is followed by years two and three at a single campus. Following this, the students undertake years four and five at one of three geographically separate campuses. The final year is a Trainee Intern Year, with students spread across many different healthcare locations, working as members of healthcare teams.

The fifth year at one of these campuses includes a five-week module in General Medicine and Subspecialties (respiratory, cardiology, oncology, nephrology). Six groups of students rotate through this module each year with approximately 14 students per rotation. The module includes clinical education in a variety of contexts including wards, outpatients, procedure clinics, emergency department and medical assessment unit. In addition there is a tutorial programme to complement the learning in context. Assessment for the module includes a multiple choice question (MCQ) examination, an Observed Structured Clinical Examination, case reports, peer review, engagement in a variety learning opportunities, and observations of professional behaviour.

### Participants

The students whose assessment responses were the focus for this study had all progressed through to year five at one campus. Each year, it is assumed likely that the level of performance within the group will range from ‘just below the standard’ required to exit year five, through to ‘excellent’ for the year of study. Decisions to halt progress are usually made at the end of the year to allow students time to make up for any deficiencies in their assessed performance during the course of the year.

### Question pool

The MCQ examinations used in this research comprised 20 single best answer multiple-choice questions that include certainty in, and safeness of, responses. Six examinations each of 20 questions drawn from a pool of 43 questions were developed. Each question consisted of a clinical scenario and a related query. Each question had 10 possible response choices. The tenth response choice was the same for each question: “I do not know, so would ask someone or seek a reference”.

### Question review

A panel of six practising clinicians developed and reviewed the questions focussing on content and correct response. Once finalised, the six clinicians also indicated the degree of safeness of each of the incorrect options. Each incorrect option was independently judged by the clinicians as ‘not unsafe’, ‘low unsafe’, ‘moderately unsafe’ or ‘highly unsafe’ for a starting year 6 student. This range of options including ‘not unsafe’ reflects the range that will occur in practice [8]. The median for these four possible category responses from the clinicians was taken as the safeness level. The median was used because this is ordinal data and enabled results to be arranged simply on the scale from ‘not unsafe’ to ‘high unsafe’. If the median was between categories, the more unsafe one was used.

The 43 questions each had 8 incorrect responses, giving a total of 344. All incorrect response were reviewed but not by all panellists, the mean number of reviews per incorrect response was 4.2 (range 2-6). Kappa statistic for agreement in these judgments was estimated as 0.29 (using Stata/IC 14.1, StataCorp LP, College Station, TX, USA).

Of the 344 incorrect responses: 135 were deemed ‘not unsafe’, 127 were ‘low level unsafe’, 68 were ‘moderately unsafe’ and 14 were classified as ‘highly unsafe’. Hence, the random chance of students choosing any response was: correct 0.10; ‘not unsafe’ 0.31; ‘low unsafe’ 0.30; ‘moderately unsafe’ 0.16; ‘highly unsafe’ 0.03; and ‘don’t know’ 0.10.

Judgments by these clinicians were also used to standard-set the questions using the modified Angoff method [35], with each clinician estimating the proportions of students with performance at a minimum standard to enter year 6, who would get each question correct.

### Question selection

The 20 questions selected for each examination for each module group varied for each group within a year, but each was blueprinted to ensure a similar mix of clinical scenarios and to ensure that the standard of minimum performance to progress to year 6, defined using the modified Angoff method, was 12/20 correct.

### Question delivery

The assessment process was explained to the students by a member of the academic staff, at the start of the module. In addition, there was written information and practice questions on the students’ learning management system (LMS).

Questions were delivered using the LMS. The front page of the site included a reminder to the students of the descriptors for levels of certainty (Table 1). These descriptors were chosen as they are authentic to reflection-in-action of daily practice [1]. To respond to the questions, the students clicked one check box for their response choice and, unless the “don’t know” response was selected (corresponding to no certainty), a second check box was provided in which they could indicate their level of certainty (low, moderate or high). The time allowed was 40 min to answer 20 questions.

**Table 1:** Descriptors of certainty

	Certainty			
	None	Low	Moderate	High
Related to knowledge	The student has no idea of correct response and any response would be a guess.	The student has no clear idea of correct response but has some knowledge on the subject. Any response would be based on limited information.	The student has a reasonable idea of correct response on a basis of moderate knowledge on the subject. Any response would be based on sufficient information	The student is certain of correct response on a basis of detailed knowledge on the subject. Any response would not be a guess.
Related to practice	The student would need to consult a colleague or references prior to considering any response.	The student would need to consult a colleague or references but would be able to give a response first.	The student would need to consult a colleague or references to confirm the correctness of the response.	The student would have no need to consult a colleague or reference.

Feedback sent to students was in the form of a matrix, displaying for each question, the question topic, the certainty of their responses and safeness. In addition, there was specific notice drawn to the topics of those questions with responses higher in certainty and unsafeness [32].

### Use of assessment results: what the students were told

In addition to providing feedback to the students for their use in future learning, the response pattern was used for a summative purpose to inform pass/fail decisions for the module. Information of certainty in, and safeness of, responses was used to make these decisions not based solely on number correct. The students were aware of the pass/fail criteria in advance.

The following constituted a fail:

- More than 2/20 highly or moderately unsafe responses that were held with high certainty, irrespective of the number correct.
- A score of fewer than 8/20 correct.

The following constituted a pass:

- A score of 12/20 or more correct.

The following constituted an excellent pass:

- A score more than 15/20 correct, with 3 or more of correct responses held with high certainty would constitute a pass noted as ‘excellent’ performance.

A score of 8-11/20 could be tolerated, allowing the student time to improve, unless information from other assessments indicated more global substandard performance.

These levels of tolerance and classification were decided by a consensus discussion by module staff.

### Statistical analysis

With regard to general data analysis, internal consistency for correct responses was calculated, irrespective of certainty. From this and the standard deviation of total correct for each student, the standard error of measurement for number correct was calculated [36].

To address the research questions, the following statistical analyses were undertaken.

For each research question a mixed model logistic regression analysis was used which controlled for module group and student by including those as random terms. To examine the relationship between being correct and response certainty the log of the odds of being correct (vs not) was modelled with terms for level of certainty (as categories). For the relationship between a potentially adverse consequence and response certainty both the log of the odds of any unsafe response (vs not) and the log of the odds of a moderately or highly unsafe response (vs low or not unsafe) were modelled with terms for level of certainty (as categories). The relationships with unsafe responses were examined for all (correct and incorrect) responses and among incorrect responses. The models included random terms for level of certainty and student module group with students as subjects nested in the module groups. To test whether these relationships with certainty and consequence vary with score groupings additional models, for each outcome, were analysed with terms for these score groups (as categories), and their interactions with level of certainty, were added to the models. When interactions were significant, whether there were differences between levels of certainty within each group, and whether differences between levels of certainty were different between groups, were tested. A *p*-value <0.05 was considered significant.

When there were significant differences among the levels of certainty, comparisons between levels of certainty (three comparisons) were adjusted for multiple comparisons with the Holm–Bonferroni method. When there was a significant interaction, tests for any differences between levels of certainty (four comparisons, one for each score group), comparisons between levels of certainty within each significant score group (three comparisons), and comparisons of differences between level of certainty between score groups (six comparisons, between pairs of score groups) were also adjusted for multiple comparisons with the Holm–Bonferroni method. When these later tests were significant, differences between levels of certainty (three comparisons) were also adjusted. Each adjustment was made independently. The adjusted criteria for considering *p*-values significant were 0.05/(the number of comparisons) for the smallest *p*-value, 0.05/(the number of comparison – 1) for the next smallest, and similarly for others with 0.05/(the number of comparison – the number already considered). Once a *p*-value was considered not significant, no further *p*-values were examined.

The glimmix procedure of SAS 9.4 (SAS Institute Inc., Cary, NC, USA) was used, with Laplace’s method and the between-subject and within-subject method for the denominator degrees of freedom.

The scores of 8/20, 12/20 and 16/20, which had been used for decision-making thresholds, having been generated from the Angoff procedure and consensus discussion amongst module staff, were used to group the students by performance in the assessment. Score groupings were:

1. low scorers (<8/20 correct)
2. below standard scorers (8-11/20 correct)
3. above standard scorers (12-15/20 correct)
4. excellent scorers (>15/20 correct).

## Results

### General data

For the assessments from the four years 2011-2014, four students’ data had to be removed from the analysis because of technical problems with question delivery on the LMS. The remaining 330 students sat the examinations with 20 students scoring in the low score group (SG1), 159 in the below standard group (SG2), 133 in the above standard group (SG3), and 18 in the excellent group (SG4).

Of the 6600 question attempts 284 (4.3%) were answered don’t know and 3747 (56.8%) were answered correctly. Of the correct responses 807 (21.5%) were low certainty, 1675 (44.7%) moderate certainty and 1265 (33.8%) high certainty. Of the 2569 incorrect responses 1407 (54.8%) were not unsafe, 986 (38.4%) lowly unsafe, 146 (5.7%) moderately unsafe and 30 (1.2%) highly unsafe, and 930 (36.2%) were low certainty, 1192 (46.4%) moderate certainty and 447 (17.4%) high certainty.

There were 4 responses held with high certainty deemed to be highly unsafe, each from a different student, one of whom was in the SG4, two from SG2 and one from SG1, and no student had 3 or more highly or moderately unsafe responses that were held with high certainty.

Each test consisted of 20 MCQ questions, with limited internal consistency (α) for correctness, and SEM for total correct between 8 and 10%, as shown in Table 2.

**Table 2:** Measures of internal consistency of correct responses and variance in total correct across the 6 tests

Test	Number of students	α for correct responses	SD for total correct	SEM for total correct
1	56	0.21	2.17	1.93
2	56	0.42	2.47	1.89
3	56	0.43	2.29	1.73
4	54	0.43	2.33	1.76
5	52	0.08	1.90	1.82
6	56	0.68	3.27	1.84

### Relationship between levels of certainty and the odds of correctness

Students were more likely to give a correct response with increasing certainty overall and from low to moderate and from moderate to high certainty. The odds ratio (OR) for being correct was: 1.7 (95%CI 1.5–2.0) for moderate vs low certainty; 2.1 (95%CI 1.9–2.5) high vs moderate certainty; and 3.7 (95%CI 3.1–4.3) for high vs low certainty (all *p* < 0.0001).

The differences among levels of certainty were significantly different between score groups (interaction of score group and level of certainty F = 3.6 degrees of freedom df = 6570 *p* = 0.002) (Table 3). The differences in odds of being correct among levels of certainty for all score groups were significantly different (*p* = 0.045 for SG1 and *p* < 0.0001 for each of the other groups). After adjusting for multiple comparisons, there were no significant differences between levels of certainty for SG1. There were significant differences between all levels of certainty for the other score groups, except for SG4 between moderate and high certainty (*p* = 0.39).

**Table 3:** Odds ratio of a correct response by certainty and score group

	Score group			
	SG1 *n* = 20	SG2 *n* = 159	SG3 *n* = 133	SG4 *n* = 18
Low certainty	1.0	1.6 (1.0–2.4) 0.036	3.4 (2.2–5.3) <0.0001	5.7 (3.1–10.5) <0.0001
Moderate certainty	1.8 (1.1–3.1) 0.032	2.5 (1.6–3.7) <0.0001	5.5 (3.6–8.4) <0.0001	19.9 (10.8–36.6) <0.0001
High certainty	1.9 (1.1–3.5) 0.027	6.3 (4.1–9.8) <0.0001	10.6 (6.8–16.7) <0.0001	14.4 (7.2–29.1) <0.0001

Odds ratio, (95% confidence interval), *p*-value

After adjusting for multiple comparisons for differences between groups, the ORs for SG2 were significantly different to SG4 (*p* = 0.003). Other differences between score groups were not significant, SG1 and SG2, SG3 and SG4 (*p* = 0.010, *p* = 0.14 and *p* = 0.29 respectively), SG2 and SG3 (*p* = 0.14) and SG3 and SG4 (*p* = 0.018).

### Relationship between levels of certainty and the odds of unsafeness among all (correct and incorrect) responses

Students were less likely to give an unsafe response with moderate or high certainty than low certainty. The OR for moderate certainty vs low was 0.71 (95%CI 0.61–0.83; *p* < 0.0001), high vs low 0.65 (95%CI 0.55–0.78; *p* < 0.0001).

The differences among levels of certainty were significantly different between score groups (interaction of score group and level of certainty F = 2.7 df = 6570 *p* = 0.013) (Table 4). There were significant differences in the odds of an unsafe response between levels of certainty for SG2 (*p* < 0.0001, where all differences were significant), but not the other groups of scorers (SG1 *p* = 0.095, SG3 *p* = 0.20, SG4 *p* = 0.25).

**Table 4:** Odds ratio of an unsafe response among all (correct and incorrect) responses by certainty and score group

	Score group			
	SG1 *n* = 20	SG2 *n* = 159	SG3 *n* = 133	SG4 *n* = 18
Low certainty	1.0	0.73 (0.49–1.11) 0.14	0.38 (0.24–0.58) <0.0001	0.19 (0.08–0.43) <0.0001
Moderate certainty	0.55 (0.32–0.97) 0.038	0.52 (0.34–0.77) 0.001	0.30 (0.20–0.45) <0.0001	0.12 (0.06–0.25) <0.0001
High certainty	0.90 (0.50–1.63) 0.73	0.37 (0.24–0.56) <0.0001	0.32 (0.21–0.49) <0.0001	0.26 (0.12–0.55) 0.0004

Odds ratio, (95% confidence interval), *p*-value

After adjusting for multiple comparisons, the differences in OR between groups were not significant, SG1 compared to SG2, SG3 and SG4 (*p* = 0.041, *p* = 0.37 and *p* = 0.75 respectively), SG2 compared to SG3 (*p* = 0.019) and SG4 (*p* = 0.039), and SG3 and SG4 (*p* = 0.33).

### Relationship between levels of certainty and the odds of an unsafe response among incorrect responses

When students gave an incorrect response, questions answered with high certainty were more likely to be unsafe than those answered with low certainty (OR 2.2; 95%CI 1.7–2.8; *p* < 0.0001) or moderate certainty (OR 2.3; 95%CI 1.8–2.8; *p* < 0.0001).

The differences among levels of certainty were significantly different between score groups (interaction of score group and level of certainty F = 3.4 df = 6446 *p* = 0.002) (Table 5). There were significant differences among levels of certainty for SG2 (*p* = 0.0007), SG3 (*p* < 0.0001) and SG4 (*p* = 0.014), but not for SG1 (*p* = 0.13). For SG2 and SG3 high certainty was significantly more likely to be associated with an unsafe response than moderate certainty (OR 1.8; 95%CI 1.3–2.4; *p* = 0.0001; OR 3.6; 95%CI 2.4–5.6 *p* < 0.0001 respectively). For SG2, SG3 and SG4 scorers high certainty was significantly more likely to be associated with an unsafe response than low certainty (OR1.5; 95%CI 1.1–2.1; *p* = 0.006; OR 4.3; 95%CI 2.8–6.6; *p* < 0.0001; OR 9.2; 95%CI 2.0–42.0; *p* = 0.004 respectively).

**Table 5:** Odds ratio of an unsafe response among incorrect responses by certainty and score group

	Score group			
	SG1 *n* = 20	SG2 *n* = 159	SG3 *n* = 133	SG4 *n* = 18
Low certainty	1.0	0.9 (0.6–1.4) 0.62	0.7 (0.4–1.1) 0.12	0.4 (0.2–1.1) 0.083
Moderate certainty	0.7 (0.4–1.3) 0.22	0.8 (0.5–1.2) 0.26	0.8 (0.5–1.3) 0.34	1.2 (0.5–3.28) 0.70
High certainty	1.4 (0.7–2.8) 0.35	1.4 (0.8–2.3) 0.21	2.9 (1.6–5.1) 0.0003	4.0 (1.0–15.6) 0.043

Odds ratio, (95% confidence interval), *p*-value

After adjusting for multiple comparisons, SG2 respond differently in terms of unsafe, with incorrect, with increasing certainty compared to SG3 (*p* = 0.001). SG1 were not significantly different from SG2 (*p* = 0.76), SG3 (*p* = 0.027) or SG4 (*p* = 0.038). SG2 were not significantly different from SG4 (*p* = 0.043). SG3 and SG4 were not significantly different (*p* = 0.35). The ORs of an unsafe response for high versus low certainty were significantly higher for SG3 than SG2 (*p* = 0.0002). The OR for high versus moderate certainty for SG3 (OR 3.6) was significantly higher than SG2 (OR 1.8) (*p* = 0.007).

### Relationship between levels of certainty the odds of moderate or highly unsafe responses among all (correct and incorrect) responses

Highly unsafe responses have the potential to be most important with regards to adverse consequences. However, the number of highly unsafe responses chosen was only 30/6600. Therefore to analyse for those unsafe responses that were likely to have the greatest adverse consequences, those deemed moderate and highly unsafe were combined.

Students were less likely to give a moderately or highly unsafe response with increasing certainty. The odds ratio for moderate certainty vs low was 0.53 (95%CI 0.36–0.77; *p* = 0.0009), high vs low 0.35 (95%CI 0.23–0.53; *p* < 0.0001) and high vs moderate 0.66 (95%CI 0.46–0.93; *p* = 0.019).

The differences between levels of certainty were not significantly different between score groups (interaction of score group and level of certainty F = 1.4 df = 6570 *p* = 0.21) (Table 6).

**Table 6:** Odds ratio of a moderately or highly (clinically significant) unsafe response among all (correct and incorrect) responses by certainty and score group

	Score group			
	SG1 *n* = 20	SG2 *n* = 159	SG3 *n* = 133	SG4 *n* = 18
Low certainty	1.0	0.61 (0.30–1.25) 0.18	0.33 (0.15–0.72) 0.005	0.12 (0.02–0.96) 0.046
Moderate certainty	0.33 (0.10–1.08) 0.067	0.40 (0.20–0.82) 0.012	0.15 (0.06–0.33) <0.0001	0.06 (0.01–0.47) 0.008
High certainty	0.79 (0.27–2.25) 0.65	0.15 (0.06–0.37) <0.0001	0.14 (0.05–0.35) <0.0001	0.12 (0.01–0.94) 0.044

Odds ratio, (95% confidence interval), *p*-value

### Relationship between levels of certainty the odds of moderate or highly unsafe responses among incorrect responses

When students gave an incorrect response, there was no significant difference in the chance of a moderately or highly unsafe response between levels of certainty (F = 2.3 df = 2452 *p* = 0.098). The odds ratio for moderate certainty vs low was 0.70 (95%CI 0.50–0.98; *p* = 0.038), high vs low 0.74 (95%CI 0.47–1.16; *p* = 0.19) and high vs moderate 1.06 (95%CI 0.67–1.66; *p* = 0.82).

The differences between levels of certainty were not significantly different between the score groups (interaction of score group and level of certainty F = 0.8 df = 6446 *p* = 0.54) (Table 7).

**Table 7:** Odds ratio of a moderately or highly (clinically significant) unsafe response *among incorrect responses* by certainty and score group

	Scorer group			
	SG1 *n* = 20	SG2 *n* = 159	SG3 *n* = 133	SG4 *n* = 18
Low certainty	1.0	0.71 (0.34–1.47) 0.36	0.56 (0.25–1.23) 0.15	0.27 (0.03–2.26) 0.23
Moderate certainty	0.39 (0.12–1.32) 0.13	0.56 (0.27–1.16) 0.12	0.32 (0.14–0.74) 0.008	0.37 (0.04–3.08) 0.36
High certainty	1.01 (0.34–2.98) 0.99	0.38 (0.16–0.92) 0.032	0.52 (0.20–1.33) 0.17	0.57 (0.07–4.86) 0.61

Odds ratio, (95% confidence interval), *p*-value

## Discussion

Within the cohort as a whole and the four performance groups, students had greater odds of correct responses as certainty increased, except for the lowest (SG1) and highest (SG4) score groups where there was no significant difference between moderate and high certainty. In addition, within the cohort as a whole and the below standard score (SG2) groups, students had lower odds of incorrect and unsafe responses for moderate and high certainty. Such findings are to be expected and are reassuring for self-monitoring in practice as markers of appropriate certainty and consideration of consequence.

When a student response was incorrect, the odds of such responses being unsafe were higher with high certainty. This was seen in all groups except the lowest performers. If this were true in practice, when practitioners happen to be incorrect, if they are also more certain, then they are also more likely to be unsafe, clearly a situation that could compound circumstances towards patient harm. One reassurance was that for those unsafe responses that are more likely to be clinically significant (moderate or highly unsafe), the odds of such responses amongst incorrect responses did not significantly vary with certainty.

The low numbers in SG1 and SG4 do mean that for some analyses the failure to demonstrate a statistically significant difference may be due to low power. Likewise low numbers of responses may limit the ability to detect statistically significant differences when analysing higher levels of unsafe responses.

### Appropriate certainty from below standard scorers

The lower scoring groups (SG1 and SG2) did show evidence of accurate self-monitoring. The lowest scoring group (SG1) had increasing odds of being correct between low certainty and moderate certainty and between low certainty and high certainty, but there was no significant difference between moderate and high certainty. The below standard group (SG2) demonstrated increasing odds of being correct with increasing certainty across all levels of certainty. In part, this confirms some recent literature [7, 37], but is contrary to a body of general literature [19, 21, 38–42], and healthcare literature [43–45] which finds that those of lesser skill are also less aware of their underperformance. The reasons our results may differ from other literature may relate to the manner in which certainty was assessed. Unlike those with contrary results, the current study used certainty descriptors that refer to seeking assistance; this is more authentic to practice [46]. Other researchers have used probabilistic predictions for certainty, such as estimating a probability that a response is correct [6, 47], a format that may not be appropriate in all contexts [48]. Instead, written descriptors for certainty may be preferable in some situations [49], such as in complex healthcare decisions [46]. In other words, clinicians do not commonly ask if they are 30% likely to be correct or not. Instead they ask themselves if they need extra information or help. The wording of certainty descriptors can alter responses and therefore meaning [50]. In the current study, certainty reflects self-assessment of performance done ‘in the moment’ rather than retrospectively [51], and is reflection-in-action authentic to daily practice [1], recognising the role of external cognitive support [8]. An assessment that addresses reflection-in-action of daily practice could be more important for ensuring safe and effective clinical performance [1]. Other means of assessing question by question self-monitoring have included a linear confidence scale [6] and flagging questions [7]. Exploring a student’s certainty based on descriptors is preferable to a student’s self-assessment relative to peers or estimating their mark [1].

### Incorrect and unsafe with increasing certainty

The higher odds for unsafe responses when incorrect as certainty increased is consistent with our prior research findings using a similar format but in a non-summative assessment setting [30]. Replicating aspects of previous findings in different student groups increases the generalisability of the results. Previously, others reported that the number of unsafe responses is proportional to the numbers of incorrect responses, and therefore this additional metric adds nothing [25, 27]. However, all previous investigations have been retrospective, where the candidates were not given any scoring disincentive for responding unsafely [25–27].

Why might some students (SG2, SG3 and SG4) have a greater proportion of incorrect responses that are unsafe when they are highly certain? There are several possible explanations, which are not mutually exclusive.

First, the students have only experienced number correct scoring with feedback that related to correctness or not, rather than to safety [30]. Feedback that is verification without elaboration is less useful in guiding learning [52]. Could it be that simply scoring on the number correct, which these students had experienced, encourages a “guess when unsure” strategy, despite this being inappropriate in clinical practice [29]? The students’ typical assessment behaviour could therefore be towards giving responses that have a chance of being correct because safety of incorrect responses has never been raised as an issue.

Second, students whose ability levels are close to threshold (e.g. pass and excellent) may be aware of this and subsequently would be more likely to take risks to increase their scores, and within these increasing responses there are increasing unsafe responses. This might lead to increasing unsafe responses with higher certainty.

Third, it is possible that knowing what to do is learnt before knowing what not to do. Knowing what not to do can be termed negative knowledge [53], and in the current study, this would be awareness of responses to avoid because of unsafeness. In the path from novice to expert, negative knowledge is gained by experience, rather than from standard learning resources, such as textbooks. The development of negative knowledge, selecting choices that are less likely to lead to a negative outcome may contribute to the increasing certainty of experts. However, those who are not yet experts, may not have had sufficient experience to develop negative knowledge with appropriate certainty. These learners still choose unsafe responses with higher certainty.

Fourth, it has been shown that for some people, as performance and certainty increase, they are more likely to take risks [54]. Although this has not been shown for those in healthcare clinical practice or students specifically, if this was a contributory factor, this would be a significant finding with regard to training and thus requires further exploration. Increasing ability may make some errors more likely, but the increase in ability is also associated with better error detection and correction [8]. In other words, ability and self-awareness may not increase in parallel – if a student’s improvement in self-awareness lagged behind their improvement in ability, then they could go through a stage of increased ability and low self-awareness before reaching a stage of increased ability and appropriate self-awareness, akin to “a little knowledge is a dangerous thing”.

Lastly, some doctors, and therefore possibly those who are in training, will have a level of certainty in their applied practice that is greater than supported by their ability. This may be due to doctors not disclosing, and therefore ignoring, uncertainty, so perceived certainty is increased [55]. There are many heuristics and biases in clinical practice that normally function to aid clinical practice, appropriately increasing certainty, but these can at times lead to incorrect decisions being made with undue certainty [56].

The fact that responses with the greatest potential for adverse consequence (moderately and highly unsafe) were not different across groups and levels of certainty could be seen as reassuring, but may reflect the low numbers of such responses with higher certainty.

### High certainty in highly unsafe responses

There were only four responses that were deemed incorrect and highly unsafe held with high certainty, one each from four students. These are rare events and if they occurred in practice would be important to recognise [8], but may have been due to keyboard error, rather than cognitive or metacognitive error. The problem of reliably assessing for rare events, such as unsafe responses, has been raised [27], and it remains an important issue. It is possible that multiple pieces of information gathered over a more prolonged time, or a failure of a student’s decision-making behaviours to change even in the light of feedback, might add to more robust information.

### Use of certainty safety responses

No person or system is perfect. Deviations from optimal acceptable practice will occur, but only some lead to adverse outcomes and patient harm [8, 16]. This can range from no clinically significant consequence to fatality [57]. Systems and staff training are in place to minimise the frequency and consequences of decisions that are deviations from optimal acceptable practice [8, 58]. Zero tolerance of deviations from optimal acceptable practice, adverse outcomes and patient harm is overly optimistic, but reducing the degree of harm from adverse events is highly desirable [8, 16, 59]. Therefore, tolerance of deviations will vary dependant on the context and what other safeguards in place [60]. Even if no clinically significant adverse event or patient harm occurs these should not be ignored but can be considered as learning opportunities [8, 58]. Therefore, when using the certainty in and safety of responses in assessment of medical students, rules regarding tolerance are required. For this assessment, these were developed by consensus amongst staff.

We have shown that considering certainty in and unsafeness of responses can add to the information gained about medical students. Specifically, such a scoring scheme may raise awareness amongst the students of decision making certainty and consequence in clinical practice. It will also reinforce seeking external support rather than guessing as appropriate practice.

The information gained regarding student ability adds to that obtained just from numbers of correct responses. Therefore, this information can be included in summative decision making with a level of tolerance for deviations from optimal acceptable practice [8]. A student may score above the pass threshold for number correct and yet still be deemed not to have passed if they displayed higher certainty in responses with higher unsafeness. If a student is close to the decision point, additional information may be needed as to whether they are safe to continue learning to improve.

The data gained from these question types could be used to provide higher quality feedback to students by including elements of metacognition, such as gauging appropriate levels of certainty, and safeness [52]. In the assessment described in this study, the feedback in terms of certainty and safety was presented specific to the question. In this way, by using these descriptors, and safety, the context specific nature of certainty was recognised [1, 9].

### Limitations

The number of questions in the test was limited, given it was covering all the various aspects of the module [61]. This led to the relatively low measures of internal consistency and high standard errors of measurement. If these are to be considered as a stand-alone test with high-stakes decisions an increase in question numbers will be required.

The low scoring (SG1) and excellent groups (SG4) had relatively small numbers of students which may limit the ability to detect statistically significant differences within and between these groups and resulted in wide confidence intervals for these comparisons. However, the below standard (SG2) and above standard group (SG3) were of larger sizes meaning that effects present in one of these and not the other is unlikely to be due to sampling.

Another limitation of the study relates to whether these measures truly reflect the degree of certainty or consideration of consequence for the students. There is no comparison with other measures or other assessments. Although the assessment did have some summative consequences, it cannot be extrapolated that this would reflect current or future practice.

Are the unsafe responses truly unsafe? Basing the safety classification on clinical outcome data from authentic practice would be ideal, such data are unlikely to be available for all the distractors [62]. However, we are reassured in that safeness was classified by the judgements of a review panel, a method consistent with previous research defining dangerous responses from the majority decision of a reviewing panel [26, 27]. However, this method showed kappa for these judgments was only fair. Although a weighted kappa may have been higher, for higher stakes assessment a larger number of reviewers, perhaps augmented by discourse, for each incorrect response would be required.

### Future directions

Although this research produced information on the cohort as a whole and groups within the cohort, detailed information about the responses of individuals and the rationale for these was not identified. The question format used in the assessment may lead to the identification of those with risk-taking response patterns. As such, correlation with other assessment results and, if possible, assessment of practice, would provide supportive evidence for this identification. This study was undertaken with a single assessment with a small number of questions covering limited content, so repeat assessments with more questions covering broader content would add to these findings. Repeat assessments would also highlight any changes in response patterns resulting from feedback, which might highlight a group of higher risk responders whose patterns do not change with feedback.

## Conclusions

1. Groups of students, classified by performance, demonstrate differing degrees of appropriate certainty and consideration of consequence. These metrics may be independent.
2. Even students with the below standard scores demonstrate increasing correctness with increasing certainty.
3. Potentially unsafe responses occur at all degrees of certainty for students in all performance groupings. Apart from those scoring lowest, students had incorrect responses that were more likely unsafe when they had high certainty.
4. We suggest that measures of certainty and consequence will add to assessment, potentially identifying those whose practice might include inappropriate certainty in unsafe actions.
